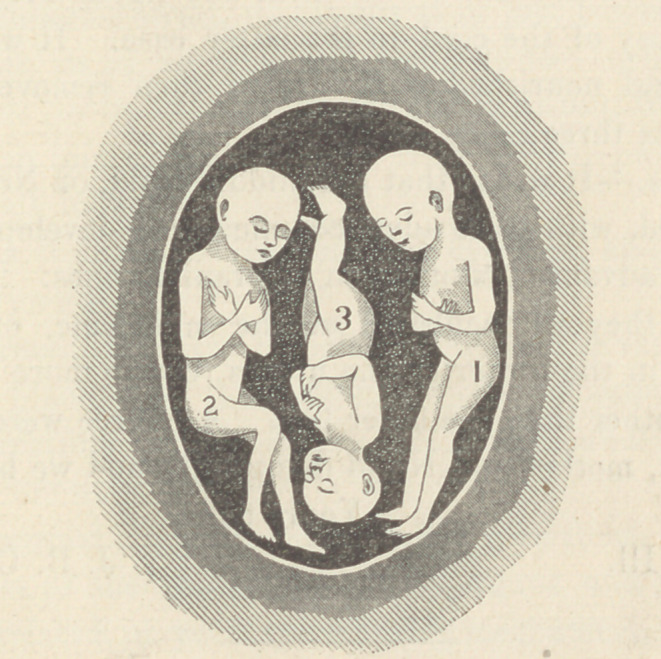# A Case of Triplets

**Published:** 1880-05

**Authors:** J. B. Crandall

**Affiliations:** Sterling, Ill.


### Clinical Reports.

### NOTES FROM PRIVATE PRACTICE.

### ARTICLE VII.

*A Case of Triplets.*

Mrs. S., wife of a mechanic in moderate circumstances, ceased to menstruate the last time on August 17, 1879. Complained of labor pains on the 6th of April, 1880.

I was called about noon; she was having light pains. I made an examination; found head presentation. The os uteri not being sufficiently dilated, I gave a powder of pulv. doveri, to be taken on the evening of the 6th. Was called about two o'clock a. m. Her pains were light and came on once every ten to fifteen minutes, till about one o'clock p. m., then the uterus being well dilated, I ordered a full dose of fluid extract of ergot, to increase the force of the pains.

No. 1 was born at half past one, with the natural head presentation. I ruptured the sac and a moderate amount of amniotic fluid escaped. The child was in appearance about seven and one half months poorly nourished; bones of the skull soft, and did not show much vitality, never cried or took a full inspiration, only gasped a few times.

No. 3, as you will see in the cut, was delivered by crowding up the breech and grasping the feet. I delivered it in about fifteen minutes after the first. This child looked different in contour, was not so long as the first, but cried lustily; showed good lung and muscular development, and weighed about one pound more than the others.

I then assured the good mother that her trouble would soon be over. But by placing my hand upon the abdomen to assist in the removal of the afterbirth, to my surprise I found another child within the uterus with a head presentation safely ensconced upon the left side. I ruptured the membrane and a large quantity of amniotic fluid escaped, more than had previously escaped, and soon after the third child was born, but never breathed, and I noticed dropsy of the cord in the latter case. It was as poorly developed and nourished as No. 1. I then removed the afterbirth with its three cords.

*Comments.*—It seems that the middle child, or No. 3, was better nourished, with more advanced muscular development, larger chest, more advanced ossification of cranial bones. The question arises were they all incubated at the same time, or would the one located in the center of the uterus receive more nourishment from the mother than the other two. All three were girls.

April 9th, mother and No. 3 doing well, and we have hopes of the father. Yours truly,

Sterling, Ill. J. B. CRANDALL.

---

DR. NORMAN BRIDGE, the librarian of the Chicago Medical Press Association, has been appointed to the medical staff of the Cook County Hospital. The appointment of such a competent man is one which is regarded with universal satisfaction.

## Figures and Tables

**Figure f1:**